# Repressed Wnt Signaling Accelerates the Aging Process in Mouse Eyes

**DOI:** 10.1155/2019/7604396

**Published:** 2019-06-17

**Authors:** Yujin Zhang, Joseph Jeffrey, Fei Dong, Jianhua Zhang, Winston W.-Y. Kao, Chia-Yang Liu, Yong Yuan

**Affiliations:** ^1^School of Optometry, Indiana University, 800 East Atwater Avenue, Bloomington, IN 47405, USA; ^2^Crawley Vision Research Laboratory, Department of Ophthalmology, College of Medicine, University of Cincinnati, Cincinnati, OH, USA

## Abstract

**Purpose:**

Ocular aging is a natural process of functional decline in vision. When the process reaches a point that compromised vision affects normal daily activity, it manifests as age-related ocular diseases, such as age-related macular degeneration, cataracts, glaucoma, and pseudoexfoliation syndrome. We previously reported that repressed Wnt signaling accelerated the maturation of corneal epithelium during tissue development. Here, we explore the hypothesis that repressed Wnt signaling is associated with accelerated aging in mouse eyes.

**Methods:**

Wnt ligand antagonist secreted frizzled-related protein 1 (sFRP1) was expressed in the corneal stroma by a tissue-specific, inducible, bitransgenic system. Tissue structure was analyzed for signs of aging. Signal transduction analysis was performed to determine the cellular response to sFRP1.

**Results:**

Mouse eyes with sFRP1 expression showed signs of accelerated aging, resembling those found in pseudoexfoliation (PEX) syndrome, a known age-related disease. Specific findings include granular deposition on the surface of the anterior lens capsule, pigment loss from the anterior surface of the iris, the presence of fibrillary material in the anterior chamber, and changes in cell size (polymegethism) and shape (pleomorphism) of the corneal endothelial cells. *In vitro* studies demonstrated that sFRP1 did not inhibit Wnt5a function and that cells responded to sFRP1 and Wnt5a in a very similar manner.

**Conclusion:**

The expression of sFRP1 accelerates the aging process in mouse eyes and future studies are warranted to elucidate the underlying mechanisms.

## 1. Introduction

Aging is a natural process that affects the function of many organs, including eyes. Patients usually experience the first sign of ocular aging when they have difficulty reading fine print. This age-related condition, called presbyopia, is caused by gradual hardening of the lens [1]. Like cataracts, the defect can be corrected by corrective lens, either inside the eyes or in front of the eyes. Other age-related conditions will manifest into debilitating eye diseases, such as age-related macular degeneration, cataracts, diabetic eye disease, glaucoma, and pseudoexfoliation (PEX) syndrome. These age-related eye diseases account for most cases of permanent vision loss and blindness. Determining the mechanism behind these conditions can provide new lifestyle and treatment guidelines that delay the onset of diseases as well as treat them.

Several signaling pathways have been implicated in aging. The first one is the target of rapamycin (mTOR) pathway. Inhibition of mTOR activity with rapamycin doubled the lifespan of simple organisms like yeast. Rapamycin treatment also extended the life of mice by about 15% percent [2]. Because mTOR serves as the master nutrient and energy sensor, this may also explain the effect of calorie restriction on aging. The second pathway is the Wnt signaling pathway. Data from *C. elegans* studies indicate that Wnt signaling plays a dual role in aging. Specifically, mom-2/Wnt and cwn-2/Wnt mutants live 35% and 18% longer compared to wild-type controls. In contrast, lin-44/Wnt and egl-20/Wnt mutants live 30% and 25% shorter than their wild-type controls [3]. Further examination of the Wnt signaling pathway may provide information on the processes involved in ocular aging.

Previously, we reported that loss of canonical Wnt function during corneal development accelerated the maturation of corneal epithelium [4]. Wnt signaling is involved in organogenesis and homeostasis. There are several Wnt signaling pathways, including canonical Wnt signaling and noncanonical Wnt signaling. A delicate balance between the two Wnt signaling pathways is key to achieving normal tissue structure and function. Canonical Wnt signaling is activated through the binding of Wnt ligands (such as Wnt3a and Wnt7) to their coreceptors (LRP5 and LRP6) [5]. This releases beta-catenin from a destruction complex and subsequently allows it to drive the expression of target genes by binding to TCF/LEF transcription factors. On the other hand, noncanonical Wnt signaling is activated through the binding of Wnt ligands (mainly Wnt5a and Wnt4) to their receptors (ROR2 or VANGL2) [6]. This triggers the activation of a panel of small GTPases, such as RhoA, Rac1, and CDC42 [7]. Subsequently, Rho-associated kinase (ROCK) is activated and transduces the signal to myosin light chain to modulate cytoskeleton structure. There have been numerous findings that the two Wnt signaling pathways inhibit each other in different tissues [8, 9]. Wnt signaling has been implicated in the development and aging process of many organs. Because loss of canonical Wnt signaling, or on the other hand increased noncanonical Wnt signaling, is associated with aging [10, 11], we set out to explore if repressed canonical Wnt signaling in adult mice can accelerate the aging process.

In our study, canonical Wnt signaling was repressed by the expression of a Wnt ligand antagonist called secreted frizzled-related protein 1 (sFRP1) in the corneal stroma. In this model, we found signs consistent with pseudoexfoliation (PEX) syndrome, an age-related disease that affects the eyes and many other organs [12]. Hallmarks of PEX syndrome observed in this model include granular deposition on the surface of the anterior lens capsule, iris stromal atrophy, accumulation of fibrillary material in the anterior chamber, and disorganized ciliary zonules. Furthermore, *in vitro* findings demonstrated that sFRP1 can stimulate the activation of noncanonical Wnt signaling. These results suggest that disruption of Wnt signaling homeostasis in the eye is associated with accelerated aging.

## 2. Methods

### 2.1. Animal Experiments

To express sFRP1 in mouse corneal stroma, bitransgenic mice (KR-sFRP1) were generated by breeding keratocan/rtTA knock-in (KR/rtTA) mice [13] with tetracycline-On promoter-driven sFRP1 (Tet-O-sFRP1) [14]. Tet-O-sFRP1 mice were a gift from Dr. Thierry Couffinhal (Hospital Haut-Leveque, Pessac, France). The knock-in *Kera*^*RT*^ mice were crossed with an enhanced GFP (EGFP) reporter mouse line, *tet-o-Hist1H*_*2B*_*-EGFP* (*TH*_*2B*_*-EGFP*, Stock number: 005104; Jackson Laboratories) [15] to obtain the double transgenic mice *Kera*^*RT*^*/TH*_*2B*_*-EGFP*. Genotyping of double transgenic mice was accomplished by PCR. The knock-in *Kera*^*RT*^ mice were identified by PCR using the following primers:

- Kera-F1, 5′-TGGTGGCTTGCTTCAAGCTTCTTC-3′
- Kera-R1, 5′-TATCCAACTCACAACGTGGCACTG-3′
- Kera-R2, 5′-GGAGTCTGCACTACCAGTACTCAT-3′

The Tet-O-sFRP1 mice were genotyped with the following primers:

- SFRP1F: 5′-TGT GTC CTC CAT GTG ACA ACG AGC-3′
- SFRP1R: 5′-TGA GAT GAG TTT TTG TTC GGG C-3′

Animal care and use conformed to the ARVO Statement for the Use of Animals in Ophthalmic and Vision Research. All animal protocols were approved by the Institutional Animal Care and Use Committee (IACUC) of the University of Cincinnati. The mice used in this project were housed in AALAC-approved animal facilities within the University of Cincinnati, College of Medicine. Programs of animal husbandry, preventive medicine, and pre- and postsurgical care have been developed to assure that adequate veterinary care is provided at all times. Complete veterinary, diagnostic, and clinical support services are available.

KR-sFRP1 bitransgenic mice (*n*=8) and their single transgenic control littermates (*n*=8) were housed in the same cage. At the age of 4 months, all mice were fed with Dox chow (1 g/kg; Custom Animal Diets, Bangor, PA, USA) for 2 months. At the age of 6 months, all mice were anesthetized by intraperitoneal injection of ketamine hydrochloride (0.1 mg/gm body weight) and xylazine (0.02 mg/gm body weight) and examined by slit lamp and stereomicroscopy.

### 2.2. Slit-Lamp Examination

Slit-lamp examinations were performed on a modified Topcon slit lamp. A beam splitter (BS7030-TOPCON) was installed in front of the eyepieces. An AccuBeam Video Adapter was attached to the beam splitter. A high-definition digital camera was mounted on the Video Adapter. Animals were anesthetized and pupils were dilated. A vertical broad slit-lamp beam was placed on the pupillary margin, and still images were taken under the same beam intensity and exposure time.

### 2.3. Stereomicroscopy and Iris Angiography

Fluorescein Ak-Fluor (10%; Akorn Pharmaceuticals), diluted with sterile 1 × DPBS (final concentration 10 mg/mL), was administered by bolus injection (50 *μ*L) into the peritoneum of anesthetized mice. For iris angiography, the nondilated eyes were observed using Zeiss Axio Zoom.V16 stereo fluorescence microscope (Oberkochen, Germany).

### 2.4. Histology and Immunostaining

Enucleated eyes were fixed in Davidson's fixation overnight and dehydrated through ethanol steps. Dehydrated eyes were immersed in paraffin overnight, then embedded and cut into sections after 24 hours. The sections were deparaffinized and rehydrated in a graded ethanol series (95%, 75% ethanol, and PBS for 3 minutes each). Rehydrated slides underwent either hematoxylin and eosin (H&E) staining, periodic acid-Schiff (PAS) staining, or immunofluorescence staining. For immunofluorescence staining, an antigen retrieval step was performed by boiling the slides in citrate buffer for 10 minutes. The following antibodies were used in the study: rabbit anti-LOXL1 (Novus Biologicals, NBP182827), rabbit anti-sFRP1 (Novus Biologicals, NBP1-02432), and Cy5-labeled goat anti-rabbit IgG secondary antibody (Invitrogen, A10523). Fluorescent images were taken with a Leica confocal microscope under 63x oil objective lens. For corneal whole-mount staining, mice were euthanatized, and the eyeball was fixed in 4% paraformaldehyde in 0.1 M phosphate buffer, pH 7.4, at 4°C overnight. After removal of the iris, lens, and posterior tissues, the cornea was incubated with 0.1% Triton X-100 in PBS for 1 hour and stained with Alexa Fluor 555 Phalloidin (ThermoFisher, A34055) at room temperature and counterstained with DAPI. Images were taken in the central region of the cornea by a Leica confocal microscope. The images were analyzed by CellProfiler software [16]. Two pipelines were developed to determine the size of the cells and the stress fiber distribution within each cell.

### 2.5. Cell Culture and Adenovirus-Mediated Gene Expression

Primary human corneal stromal cells were established from a donor cornea that was not suitable for transplantation. The primary cells were maintained in SF-1 hMSC medium (United Healthcare Inc., Taiwan). Full-length mouse Wnt5a cDNA (pLNC Wnt-5aHA) was a gift from Jan Kitajewski (Addgene plasmid #18032) [17], and mouse sFRP1 cDNA XE141 sFRP-1-CS2+ was a gift from Randall Moon (Addgene plasmid #16693; http://n2t.net/addgene:16693; RRID : Addgene_16693). These cDNA constructs were used to generate the recombinant adenovirus vector according to a published protocol [18]. Another recombinant adenovirus coding GFP alone (AdGFP) was used as a control. AdGFP, AdsFRP1, and AdWnt5a viruses were amplified and purified according to the protocol.

Cells were seeded into Corning™ 96-Well Half Area High Content Imaging Film Bottom Microplate and were infected with AdGFP or AdsFRP1 after 24 hours. 72 hours after infection, the cells were fixed for 20 minutes in 1% glutaraldehyde. Cells were permeabilized with 0.2% Triton X-100 in PBS and were stained with an antibody against sFRP1 (Novus Biologicals, NBP1-02432), CHOP (Cell Signaling, 5554S), and ROR2 (Cell Signaling, 88639S). The images were taken with an epifluorescence microscope (Axioscop2, Carl Zeiss, München-Hallbergmoos, Germany) and were photographed with a digital camera system (Axiocam, Carl Zeiss).

### 2.6. Real-Time qRT-PCR

Total RNA was isolated from cultured cells with the RNeasy Mini Kit (Qiagen). 5 *μ*g of total RNA was reverse transcribed with Maxima First Strand cDNA Synthesis Kit for qRT-PCR (Fermentas). qRT-PCRs were performed using the CFX96™ real-time PCR system (Bio-Rad) operated by CFX Manager™ software. Primer sequences used in the study were as follows:

- hCHOPf: TGGATCAGTCTGGAAAAGCA
- hCHOPr: AGCCAAAATCAGAGCTGGAA
- hMMP1f: TTGTGGCCAGAAAACAGAAA
- hMMP1r: TTCGGGGAGAAGTGATGTTC

A panel of 8 housekeeping genes (Real Time Primers, Cat#HKK1) was used to normalize the expression levels.

### 2.7. Statistical Analysis

Unpaired Student's *t*-test was used to determine the statistical significance (*P* value) of the mean values for 2-sample comparisons. Values shown on the graphs represent the mean ± SD (standard derivation). A difference between average means where *P* < 0.05 was deemed to be statistically significant.

## 3. Results

### 3.1. Macroscopic Signs of Accelerated Aging

**Figure 1:**
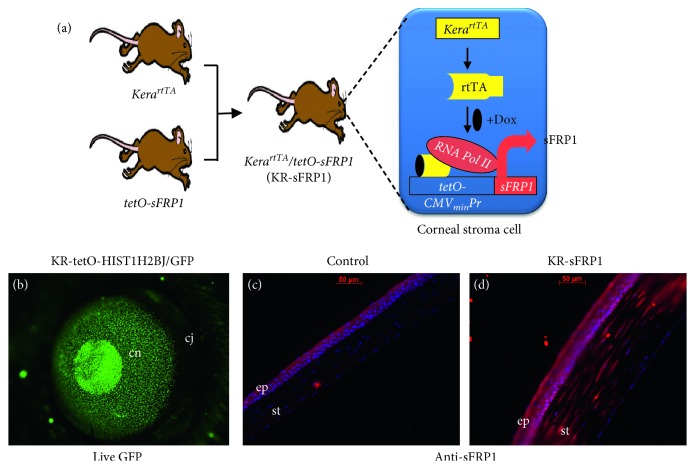
Inducible expression of sFRP1 in corneal stroma. (a) Diagram showing the generation of bitransgenic mouse strain keratocan rtTA/Tet-O-sFRP1 (KR/sFRP1). Bitransgenic mice were generated by breeding keratocan/rtTA knock-in (KR/rtTA) mice with tetracycline-On promoter-driven sFRP1 (Tet-O-sFRP1). Bitransgenic mice expressed sFRP1 in corneal stroma upon doxycycline induction. (b) Corneal stromal expression of reporter gene GFP in adult KR/GFP mice. (c, d) Immunostaining verified that only bitransgenic KR/sFRP1 mice were positive for sFRP1 in the corneal stroma. Abbreviations: cn = cornea, cj = conjunctiva, ep = epithelium, and st = stroma.

To express sFRP1 in mouse corneal stroma, bitransgenic mice were generated by breeding keratocan/rtTA knock-in (KR/rtTA) mice with tetracycline-On promoter-driven sFRP1 (Tet-O-sFRP1). Bitransgenic mice (KR/sFRP1) expressed sFRP1 in the corneal stroma upon doxycycline induction (Figure 1(a)). To verify that the target gene can be expressed in the corneal stroma in a doxycycline-dependent manner, we bred the driver mouse KR/rtTA with a reporter mouse (Tet-O-histone GFP). One week after doxycycline induction, GFP-positive cells were found in the corneas (Figure 1(b)). Two months after doxycycline induction, KR/sFRP1 mice and their control littermates were used to examine the expression of sFRP1. As shown in Figures 1(c) and 1(d), the control eyes did not express sFRP1 while there were strong sFRP1-positive cells in the corneal stroma of KR/sFRP1 eyes, confirming the expression of sFRP1 in the corneal stroma of our doxycycline-induced bitransgenic mice.

**Figure 2:**
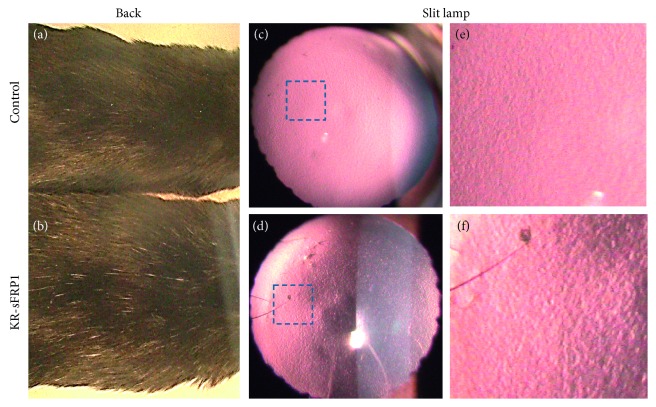
Signs of aging in KR/sFRP1 mice. (a, b) KR/sFRP1 mice had more grey hairs on the back than their control littermates, serving as a qualitative readout of aging. (c, d) KR/sFRP1 mouse eyes exhibited a rough surface on the anterior lens capsule. The granular deposits were more apparent in high-magnification images (e, f).

Two months after doxycycline induction, KR/sFRP1 mice and their control littermates were examined. Using qualitative levels of grey hair as a readout for aging, we set out to examine the amount of grey hair present on the backs of mice. At the age of six months, control mice had few grey hairs on their back (Figure 2(a), while KR/sFRP1 mice had apparently more grey hairs (Figure 2(b)). Under the slit lamp, the anterior lens capsule of the control mice appeared smooth and uniform (Figures 2(c) and 2(e)). In the KR/sFRP1 eyes, surface roughness was revealed by side illumination (Figure 2(a)). The granular characteristics presented as bright reflections and dark shadows, which were more easily appreciated in higher magnification image (Figure 2(f)). These macroscopic features are consistent with our previous findings in mouse eyes with pseudoexfoliation syndrome-like phenotypes [19].

**Figure 3:**
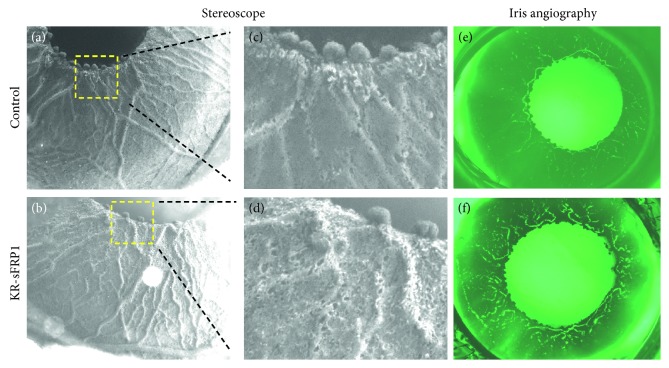
Signs of iris stromal atrophy in KR/sFRP1 mice. Overall surface texture of irises for control (a) and KR/sFRP1 (b) mice. High-magnification images revealed the difference between the two (c, d). In the control eyes, the surface area between vessels was smooth and uniform. In the KR/sFRP1 eyes, the blood vessels were bulging and tortuous. Iris angiography images of the control (e) and KR/sFRP1 (f) mice further demonstrate tortuous vessels. The atrophic findings of the KR/sFRP1 irises further demonstrate ocular findings consistent with aging.

Finally, iris atrophy was observed by live stereoscopy.  Figure 3(a) represents the normal structure of the anterior iris surface, as seen in control eyes: large radial vessels were half-buried in the stroma while networks of smaller vessels were mostly buried. The surface area between vessels was smooth and uniform. In the KR/sFRP1 eyes, the blood vessels were bulging and tortuous (Figure 3(b)). A higher degree of stromal degeneration was apparent in pupillary margins. High-magnification images showed a few small holes along the blood vessels on the iris surface of the control eyes (Figure 3(c)). In the KR/sFRP1 eyes, the size and the number of the holes increased, forming a sponge-like surface texture (Figure 3(d)). Additionally, the loss of anterior iris surface material was verified by angiography. In normal control eyes, the iris blood vessels were buried in the heavily pigmented stroma, making them invisible under fluorescent microscope (Figure 3(e)). The only visible signals were in the pupillary margin, where the vessels were exposed to the surface. In KR/sFRP1 eyes, tortuous vasculature structures were clearly visible in the middle region of the iris (Figure 3(f)). The atrophic findings of the KR/sFRP1 irises further demonstrate ocular findings consistent with aging.

### 3.2. Microscopic Signs of Accelerated Aging

**Figure 4:**
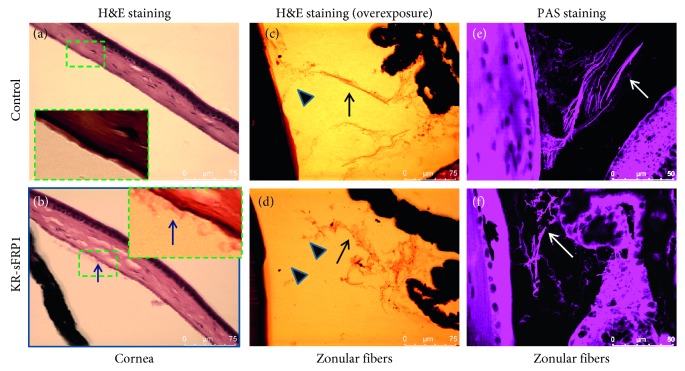
sFRP1 causes fibrillary accumulation in the anterior chamber and weakened zonules. (a, b) H&E staining of the control and KR/sFRP1 eyes. Arrow in the highlighted box indicates the fibrillary material in the anterior chamber, as evidenced in KR/sFRP1 eyes but not controls. (c) and (d) are overexposure images to reveal zonular structure (arrows). In the control eyes, the main zonular bundles were straight and compact. In the KR/sFRP1 eyes, the zonular bundles were curly, loose, disorganized, and decorated with granular material. (e) and (f) are confocal scanning images of PAS staining, which confirmed the presence of proteoglycans. Arrows indicate the differences in zonular structure.

Microscopic features of aging were examined by histological examination. The macroscopic features of the KR/sFRP1 eyes, such as granular deposition and iris atrophy, indicated signs of pseudoexfoliation (PEX) syndrome, an age-related disease. Microscopic studies were conducted to further document signs of PEX syndrome. As shown in Figure 4, the anterior chamber of the control eyes was free of aggregates (Figure 4(a)), while the KR/sFRP1 eyes contained fibrillar material (Figure 4(b)). Clumps of abnormal fibrillar material were found adhering to the apical surface of the endothelial cells and more were found floating just beneath the corneal endothelium. Another feature of PEX is the weakness of zonules [20]. The structure of zonules was revealed by overexposing the H&E images. In the control eyes, the main zonular bundles were straight and compact (Figure 4(c)). In the KR/sFRP1 eyes, the zonular bundles were curly, loose, disorganized, and decorated with granular material. Periodic acid-Schiff (PAS) staining verified the presence of proteoglycans in the zonule. Scanning of PAS-positive zonular fibers confirmed the structural difference of zonules between the control and KR/sFRP1 eyes (Figures 4(e) and 4(f)). These histological findings correlate with PEX phenotypes and indicate evidence of ocular aging.

**Figure 5:**
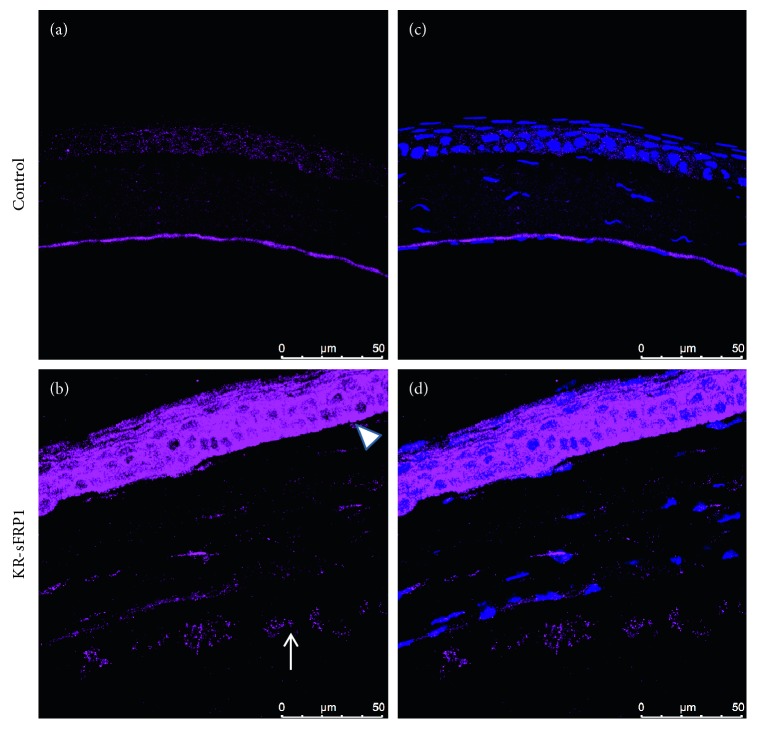
Exfoliation-like material is LOXL1-positive. LOXL1 immunostaining of control corneas (a) revealed LOXL1-positive bands on the corneal endothelium (a, c). KR/sFRP1 corneas exhibited increased LOXL1 expression in the corneal epithelium. LOXL1-positive aggregates can be found in the anterior chamber (arrow in b). These findings are highly consistent with PEX syndrome phenotypes.

Further microscopic examination was conducted to analyze the expression of lysyl oxidase-like 1 (LOXL1). LOXL1 is a member of the lysyl oxidase family of enzymes that catalyze cross-linking in the extracellular matrix (ECM). LOXL1 is a known component of ECM and therefore also of PEX material [21]. As shown in Figure 5(a), LOXL1 was weakly stained within the corneal epithelial cells in control eyes. LOXL1 formed a dense, uniform layer in Descemet's membrane (Figure 5(a)). In the KR/sFRP1 eyes, however, LOXL1 expression was increased in the corneal epithelium with a perinuclear distribution pattern (Figures 5(c) and 5(d)). The well-organized LOXL1-positive layer was not seen in Descemet's membrane. Strong positive staining was found between as well as on the apical side of the endothelial cells. The LOXL1-positive aggregates can also be found detaching from the endothelium and floating in the anterior chamber. These data further confirmed that the KR/sFRP1 eyes had some ocular features of PEX syndrome.

**Figure 6:**
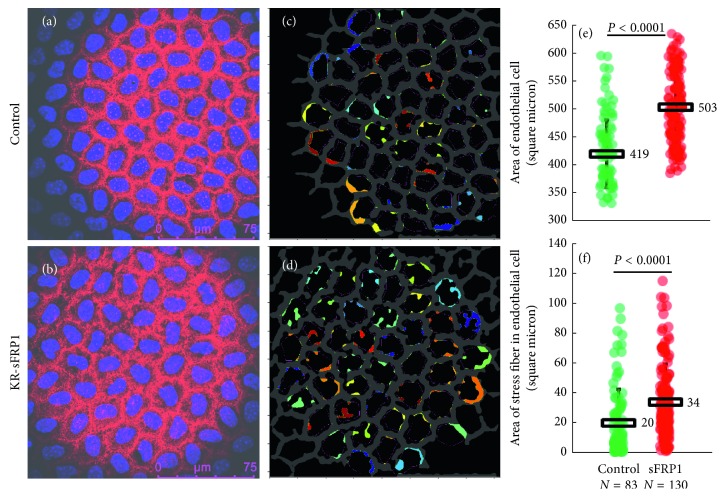
sFRP1 induces polymegethism and pleomorphism in the corneal endothelium. Original (a, b) and processed (c, d) images of the corneal endothelium of the control and KR/sFRP1 mice. Colored nodules represent the stress fibers (c, d). The structure from the control corneas was uniform with very few broken strands, while the thickness of the intercellular cytoskeleton bands varied greatly in KR/sFRP1 corneas with many broken strands. The size of the cells and the stress fibers is summarized in (e) and (f). The average size of corneal endothelial cells was 419 ± 60 square microns (*n*=83) vs. 503 ± 63 square microns (*n*=130) in KR/sFRP1 eyes (*P* < 0.0001). The average size of stress fiber per cell was also significantly increased in KR/sFRP1 eyes (20 ± 23 square microns (*n*=83) vs. 34 ± 25 square microns (*n*=130); *P* < 0.0001). These findings demonstrate polymegethism and pleomorphism consistent with aging eyes.

Another sign of aging eyes, as well as sign of PEX syndrome, is the change in cell size (polymegethism) and shape (pleomorphism) of the corneal endothelial cells [22]. Whole-mount staining was performed on two control corneas and four KR/sFRP1 corneas. The corneas were stained with phalloidin to reveal the cytoskeleton structure. In control corneas, endothelial cytoskeleton was arranged in linear circumferential strands that formed a hexagonal array (Figure 6(a)). In KR/sFRP1 corneas, circumferential strands were loosely arranged, resulting in increased bandwidth with some stress fibers extending towards the nuclei (Figure 6(b)). CellProfiler software identified the overall structure of the apical cytoskeleton networks of the endothelium and presented it as a grey “fishnet-like” structure. The structure from the control corneas was uniform with very few broken strands (Figure 6(c)), while the thickness of the intercellular cytoskeleton bands varied greatly in KR/sFRP1 corneas with many broken strands (Figure 6(d)). The colored nodules next to the intercellular cytoskeleton structure represented the stress fibers extending toward the center of the cell (Figures 6(c) and 6(d)). This stress fiber formation was more apparent in KR/sFRP1 eyes. Figures 6(e) and 6(f) summarize the size of the cells and the size of stress fibers, respectively. In control eyes, the average size of corneal endothelial cells was 419 ± 60 square microns, *n*=83. In KR/sFRP1 eyes, the average size of corneal endothelial cells was 503 ± 63 square microns, *n*=130. The difference between the means was statistically different (*P* < 0.0001). In control eyes, the average size of stress fiber per cell was 20 ± 23 square microns, *n*=83. In KR/sFRP1 eyes, the average size of stress fiber per cell was 34 ± 25 square microns, *n*=130. Again, the difference between the means was statistically different (*P* < 0.0001). These findings demonstrate polymegethism and pleomorphism consistent with aging eyes.

### 3.3. Potential Mechanisms for sFRP1-Induced Aging

**Figure 7:**
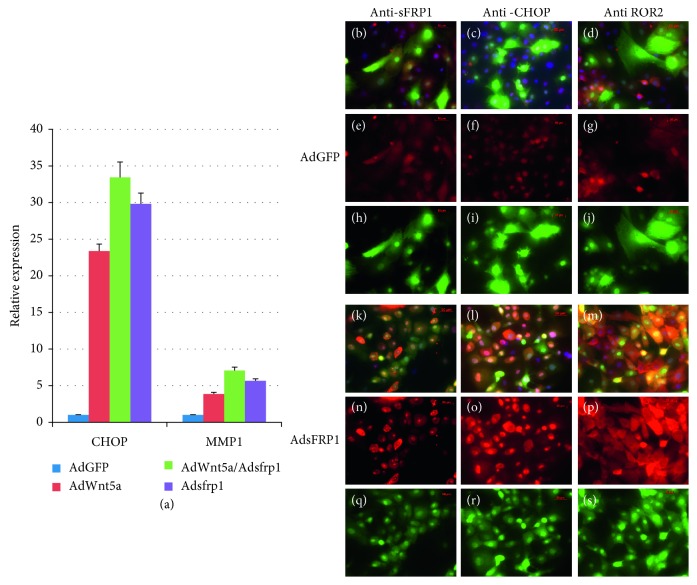
sFRP1 activates the noncanonical Wnt signaling. (a) Real-time RT-PCR demonstrated that both Wnt5a and sFRP1 can stimulate the expression of CHOP and MMP1 (red and purple bars). Stronger effects were observed when cells were treated with Wnt5a and sFRP1 together (green bar). Immunostaining demonstrated that sFRP1 stimulates the expression of CHOP and ROR2 (red channel) ((k)–(s)). Upper panels were cells infected with AdGFP control virus, and lower panels were cells infected with AdsFRP1 virus. All adenoviruses also expressed GFP. Combined channels revealed the expression of protein of interest (red) in adenovirus-infected cells (green) ((b)–(d) and (k)–(m)). Red channel signaling clearly demonstrated the increased signals from cells infected with AdsFRP1 (compare (e)–(g) with (n)–(p)). Green channel signaling revealed the adenovirus-infected cells ((h)–(j) and (q)–(s)).

sFRP1 was discovered as a naturally occurring secreted antagonist of Wnt signaling [23]. Later studies uncovered more complicated roles of sFRP1 in modulation of Wnt signaling: it acts as a biphasic modulator of Wnt signaling, counteracting Wnt-induced effects at high concentrations and promoting them at lower concentrations [24]. Because there are several Wnt signaling pathways, the balance between the canonical and noncanonical Wnt signaling is carefully regulated. Most studies show that at high concentrations, sFRP1 can inhibit canonical Wnt signaling [25, 26], which in turn favors heightened noncanonical Wnt signaling. We performed an *in vitro* study to determine if sFRP1 can also inhibit noncanonical Wnt signaling. Primary human corneal stromal cells were infected with adenovirus coding sFRP1 and Wnt5a either separately or together. Noncanonical signaling was evaluated by measuring several downstream targets: CHOP, MMP1, and ROR2 [27]. Compared with control AdGFP-infected cells, cells infected with AdWnt5a exhibited an increase in CHOP and MMP1 expression (red bar in Figure 7(a)). AdsFRP1-infected cells also exhibited increased expression of CHOP and MMP1 (purple bar in Figure 7(a)). The combination of AdWnt5a and AdsFRP1 yielded a greater degree of increased expression of CHOP and MMP1 than either treatment alone (green bar in Figure 7(a)). This result indicates that sFRP1 did not inhibit Wnt5a function and sFRP1 alone can activate noncanonical Wnt signaling. This observation was confirmed at the protein level shown in Figures 7(b)–7(s). Compared to control AdGFP-infected cells (7(b)–7(j)), AdsFRP1-infected cells exhibited higher levels of sFRP1, CHOP, and ROR2 protein (7(k)–7(s)). ROR2 is a noncanonical Wnt receptor whose expression level can be stimulated by noncanonical Wnt signaling through a feed-forward mechanism [28]. Overall, the expression of sFRP1 appears to favor noncanonical Wnt signaling, either by indirect inhibition of canonical Wnt or by direct stimulation of noncanonical Wnt signaling.

## 4. Discussion

The exact mechanisms that underlie the process of aging are currently unclear. Two theories have been proposed to be the major contributors for aging [29]. The first one is the programmed theory, and the second is the damage-related theory. The programmed theory considers aging as preprogrammed genetic events, just as were embryonic development and early childhood development. The gene expression driving these events is programmed in the DNA and is unfolded in a precisely controlled manner. Evidence supporting this theory includes the steady shortening of telomeres during the lifetime of dividing cells. Also, a decreased growth hormone/insulin-like growth factor 1 signaling pathway or nutrient-sensing mTOR signaling has been associated with increased lifespan [30]. Overactivation of mTOR signaling is also considered a major factor for stem cell depletion [31]. The damage-related theory regards aging as the consequence of a loss of cells due to accumulative damages beyond repair. We consider both theories depending on the tissue context. For tissues that can regenerate, such as skin and blood, keeping a healthy pool of stem cells is critical and therefore may relate to the programmed theory. For tissues mainly dependent on their differentiated cells to function, such as heart muscle and neurons, preventing damage-related cell loss is the best strategy in concordance with the damage-related theory.

If we consider aging as a programmed process, we can also consider it to be a developmental process destined towards self-destruction. The fate is inevitable, but the process can be prolonged and delayed. The basic premise behind our study takes into account these ideas: if a factor can accelerate the development of a tissue during early development, this factor may also accelerate the aging process later in life. This idea draws upon the facets of developmental drift [32]. According to this theory, selection pressure is the highest in early development. Evolution selects pathways that provide an early selective advantage to the animals. These pathways may also accelerate other biological processes, such as aging. We previously reported that knockout beta-catenin, a key canonical Wnt signaling mediator, from the corneal stroma accelerated the maturation of corneal epithelium [4]. Here we report signs of accelerated aging in mice expressing the Wnt antagonist sFRP1. The first sign we noticed was the increased number of grey hairs on the back of the KR/sFRP1 mice. Grey hair is a documented sign of aging, caused by oxidative stress in pigment-producing cells [33]. The KR driver mice were designed to express genes in corneal stromal cells, but a low level of expression may also occur in other tissues of neural crest origin, such as melanocytes and cartilage precursors. Because the expression pattern of the KR driver has not been fully characterized in adult mice, we can only speculate that the increased number of grey hairs is caused by sFRP1 expression in the melanocytes of hair follicles.

The signs of accelerated aging presented in the KR/sFRP1 resemble those of PEX syndrome. PEX syndrome is an age-related systemic disease characterized by the accumulation of an extracellular fibrillar material in the eyes, skin, lungs, heart, kidneys, and other organs. About 30% of patients with PEX syndrome will progress to glaucoma within 7 years [34]. Further associated clinical signs and potential complications include angle-closure glaucoma, cataracts, phacodonesis, and lens subluxation due to weakened ciliary zonules, insufficient mydriasis, saw-tooth structure of the iris pigment epithelium, peripupillary transillumination defects due to dispersion of pigment, iris stromal atrophy, iris vasculopathy associated with blood-aqueous barrier defects, and formation of posterior synechiae as well as corneal endothelial decompensation [35]. Within two months of sFRP1 expression (mice chronological age of six months), the following signs of PEX syndrome were observed: granular deposition on the surface of the anterior lens capsule, pigment loss from the anterior surface of the iris, presence of fibrillary material in the anterior chamber, and weakened ciliary zonules. We did also observe some of those signs in the much older control mice (over twelve months old, data not shown), further suggesting that sFRP1 accelerated the aging process. Currently, we do not know if the ocular alterations are directly caused by diffusible sFRP1 expressed by the keratocyte or by secondary factors due to the expression of sFRP1. Diffusible factors are critical in the homeostasis of this part of the eyes which does not have an extensive vascular network.

In addition to mTOR signaling, Wnt signaling has been implicated in the aging process [10]. The consensus is that canonical Wnt signaling delays the aging process by preserving a healthy pool of stem cell population, while noncanonical Wnt signaling accelerates the aging process by depleting the stem cell pool. The detrimental role of noncanonical Wnt signaling in aging is supported by the longevity studies in *C. elegans* of which mom-2/Wnt and cwn-2/Wnt mutants live 35% and 18% longer compared to wild-type controls. *cwn*-*2* is an ortholog of human *Wnt5a*, a known noncanonical Wnt ligand. On the other hand, lin-44/Wnt and egl-20/Wnt mutants live 30% and 25% shorter than their wild-type controls. Both lin-44 and egl-20 are orthologs of human *Wnt7*, a known canonical Wnt ligand [36]. This connection provides the foundation for the role of noncanonical Wnt signaling in the processes of ocular aging.

It is well documented that sFRP1 can inhibit canonical Wnt signaling by sequestering the Wnt ligands. If the only function of sFRP1 is binding and sequestering the Wnt ligands, it should also bind and sequester noncanonical Wnt ligands, such as Wnt5a. However, our data demonstrated that this is not the case. sFRP1 does not inhibit Wnt5a-mediated signal transduction, but rather it behaves like Wnt5a. This notion is supported by genetic studies that sFRP1 knockout mice have the same phenotypes as Wnt5a knockout mice in male sexual development [37]. Eyes expressing Wnt5a also exhibit PEX-like phenotypes just as do sFRP1 eyes [19]. So, we suggest that it is possible that sFRP1 can directly stimulate noncanonical Wnt signaling. To prove that, endogenous Wnt5a or other noncanonical Wnt ligands must be knocked out in order to rule out the possibility that sFRP1 indirectly activates noncanonical Wnt signaling by facilitating a Wnt5a feed-forward cycle. Further studies will be conducted to tease out the relationship between sFRP1 and noncanonical Wnt ligands such as Wnt5a.

In our study, we did not examine the potential important role of sFRP1 in glaucoma, another age-related eye disease. Evidence indicates that there may be a relationship between sFRP1 and glaucoma: sFRP1 is upregulated in the trabecular meshwork cells from primary open-angle glaucoma patients [38]. Also, adenovirus-mediated sFRP1 expression elicits ocular hypertension in mice [39]. Based on these reports, KR/sFRP1 should have glaucoma-related retinal ganglion cell loss. If KR/sFRP1 mice do have such retinal ganglion cell loss, it could serve as an animal model for glaucoma. We aim to further analyze the potential relationship between sFRP1 mice and glaucoma, ideally determining features that could prove beneficial to an animal model.

In summary, we found signs of accelerated aging in eyes with exogenous sFRP1 expression. The effects found were determined to be associated with increased noncanonical Wnt signaling. Further proving this connection may provide therapeutic benefits, as noncanonical Wnt inhibitors, like ROCK inhibitor Rhopressa, have been approved to ameliorate symptoms of glaucoma. Such therapeutic agents may also be very useful in preventing the accelerated aging process in eyes caused by over-activated noncanonical Wnt signaling. Further work is needed to establish sFRP1 expression as a model useful in studying phenotypes of ocular aging before any therapeutic considerations can be made. However, this study provides clues as to how noncanonical Wnt signaling in the eye may lead to accelerated ocular aging, with phenotypes consistent with other age-related ocular manifestations such as PEX syndrome.

## Data Availability

The imaging data used to support the findings of this study are included within the article. The CellProfiler Pipeline files data used to support the findings of this study are available from the corresponding author upon request.
